# 2-Amino-4-methyl­pyridinium 2-nitro­benzoate

**DOI:** 10.1107/S1600536813012919

**Published:** 2013-05-18

**Authors:** Srinivasan Muralidharan, Narayanan Elavarasu, Thothadri Srinivasan, Rengasamy Gopalakrishnan, Devadasan Velmurugan

**Affiliations:** a Department of Physics, Anna University, Chennai 600 025, India; b Centre of Advanced Study in Crystallography and Biophysics, University of Madras, Guindy Campus, Chennai 600 025, India

## Abstract

In the title mol­ecular salt, C_6_H_9_N_2_^+^·C_7_H_4_NO_4_^−^, the original pyridine N atom of 2-amino-4-methyl­pyridine is protonated and the carb­oxylic acid group of nitro­benzoic acid is deprotonated. In the crystal, the ions are linked by N—H⋯O hydrogen bonds, forming chains propagating along [001]. The chains are linked *via* C—H⋯O hydrogen bonds, forming two-dimensional networks lying parallel to the *bc* plane.

## Related literature

For related structures, see: Navarro Ranninger *et al.* (1985 ▶); Luque *et al.* (1997 ▶); Qin *et al.* (1999 ▶); Jin *et al.* (2001 ▶); Albrecht *et al.* (2003 ▶); Kvick & Noordik (1977 ▶).
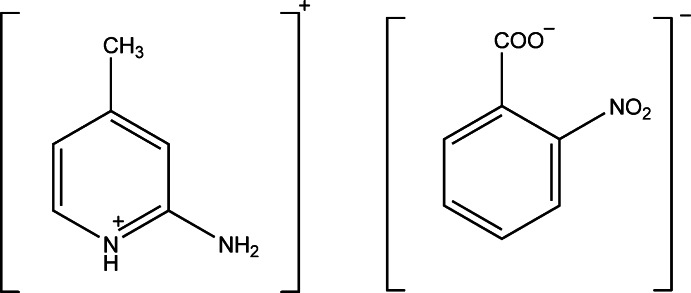


## Experimental

### 

#### Crystal data

C_6_H_9_N_2_^+^·C_7_H_4_NO_4_^−^
*M* *_r_* = 275.26
Monoclinic, 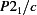

*a* = 12.2049 (3) Å
*b* = 9.8463 (2) Å
*c* = 11.5405 (2) Å
β = 107.106 (1)°
*V* = 1325.51 (5) Å^3^
*Z* = 4
Mo *K*α radiation
μ = 0.11 mm^−1^
*T* = 293 K
0.30 × 0.25 × 0.20 mm

#### Data collection

Bruker SMART APEXII area-detector diffractometer
Absorption correction: multi-scan (*SADABS*; Bruker, 2008 ▶) *T*_min_ = 0.969, *T*_max_ = 0.979
12523 measured reflections
3289 independent reflections
2644 reflections with *I* > 2σ(*I*)
*R*_int_ = 0.023

#### Refinement

*R*[*F* ^2^ > 2σ(*F* ^2^)] = 0.041
*wR*(*F* ^2^) = 0.125
*S* = 1.06
3289 reflections
195 parameters
H atoms treated by a mixture of independent and constrained refinement
Δρ_max_ = 0.26 e Å^−3^
Δρ_min_ = −0.18 e Å^−3^

### 

Data collection: *APEX2* (Bruker, 2008 ▶); cell refinement: *SAINT* (Bruker, 2008 ▶); data reduction: *SAINT*; program(s) used to solve structure: *SHELXS97* (Sheldrick, 2008 ▶); program(s) used to refine structure: *SHELXL97* (Sheldrick, 2008 ▶); molecular graphics: *ORTEP-3 for Windows* (Farrugia, 2012 ▶); software used to prepare material for publication: *SHELXL97* and *PLATON* (Spek, 2009 ▶).

## Supplementary Material

Crystal structure: contains datablock(s) global, I. DOI: 10.1107/S1600536813012919/su2590sup1.cif

Structure factors: contains datablock(s) I. DOI: 10.1107/S1600536813012919/su2590Isup2.hkl

Supplementary material file. DOI: 10.1107/S1600536813012919/su2590Isup3.cml

Additional supplementary materials:  crystallographic information; 3D view; checkCIF report

## Figures and Tables

**Table 1:** Hydrogen-bond geometry (Å, °)

*D*—H⋯*A*	*D*—H	H⋯*A*	*D*⋯*A*	*D*—H⋯*A*
N3—H3*A*⋯O4^i^	0.933 (18)	1.752 (19)	2.6746 (16)	169.5 (17)
N4—H4*A*⋯O3^i^	0.923 (19)	1.972 (19)	2.8734 (18)	164.9 (17)
N4—H4*B*⋯O4^ii^	0.871 (19)	2.033 (19)	2.8937 (16)	169.7 (18)
C11—H11⋯O3^iii^	0.93	2.58	3.3624 (16)	142

Symmetry codes: (i) 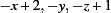; (ii) 
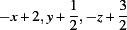
; (iii) 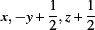.

**Fig. 1.**
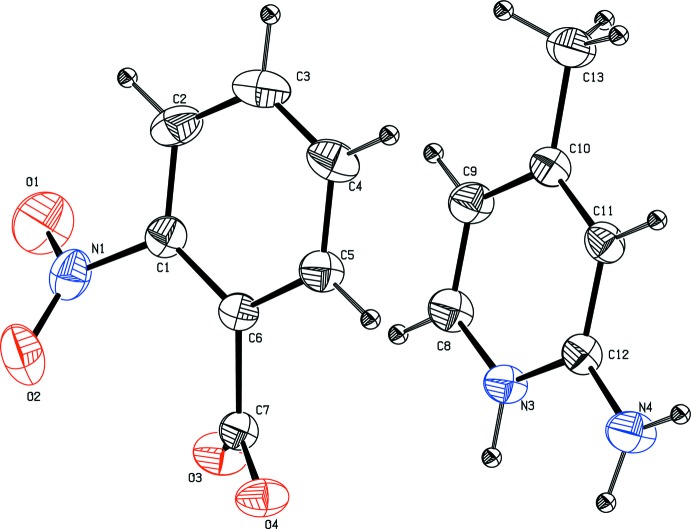
The molecular structure of the title compound, with atom labelling. Displacement ellipsoids are drawn at the 30% probability level.

**Fig. 2.**
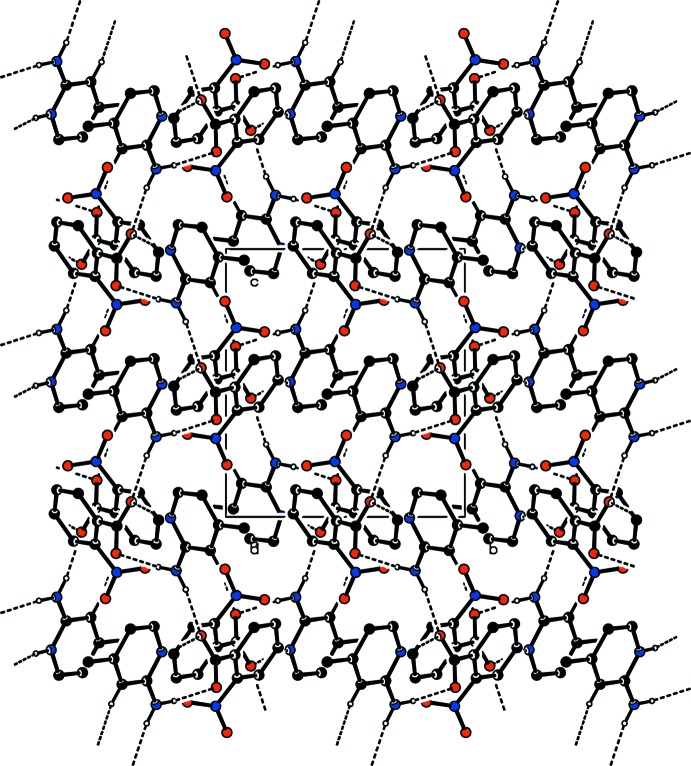
The crystal packing of the title compound viewed along the a axis. The hydrogen bonds are shown as dashed lines (see Table 1 for details; C-bound H-atoms have been omitted for clarity).

## supplementary crystallographic information

### Comment

There are numerous examples of 2-amino-substituted pyridine compounds in which the 2-aminopyridines act as neutral ligands (Navarro Ranninger *et al.*, 1985; Luque *et al.*, 1997; Qin *et al.*, 1999) or as protonated cations (Luque *et al.*, 1997; Jin *et al.*, 2001; Albrecht *et al.*, 2003). In order to study hydrogen bonding interactions in such systems, we synthesized the title salt and report herein on its crystal structure.

In the title molecular salt, Fig. 1, the pyridine N atom of 2-amino-4-methylpyridine is protonated and the carboxyl group of nitrobenzoic acid is deprotonated. The amine attached with the pyridine ring deviates by -0.0098 (15) Å. The methyl carbon atom C13 attached with the pyridine ring deviates by -0.0261 (17) Å.

In the crystal, the pyridine ring (N3,C8-C12) makes a dihedral angle of 12.13 (7)° with the nitrobenzoate ring (C1-C6). The ions are linked by N–H···O hydrogen bonds forming chains propagating along [001]; see Table 1 and Fig. 2. These chains are linked via C–H···O hydrogen bonds forming two-dimensional networks lying parallel to the bc plane (Table 1).

### Experimental

2-amino-4-methylpyridine (C_6_H_8_N_2_) and 2-nitrobenzoic acid (C_7_H_5_N_1_O_4_) were mixed in an equimolar ratio (1:1) using ethanol as solvent and stirred well. The solution was filtered into a clean beaker and optimally closed. Colourless block-like crystals were obtained by slow evaporation at room temperature in 15 days.

### Refinement

The NH and NH_2_ H atoms were located in a difference Fourier map and freely refined. The C-bound H atoms were positioned geometrically and refined using a riding model: C—H = 0.93 and 0.96 Å for CH and CH_3_ H atoms, respectively, with U_iso_(H) = 1.5U_eq_(C) for CH_3_ H atoms and = 1.2U_eq_(C) for other H atoms.

### Crystal data

**Table:**

C_6_H_9_N_2_^+^·C_7_H_4_NO_4_^−^	*F*(000) = 576
*M**_r_* = 275.26	*D*_x_ = 1.379 Mg m^−^^3^
Monoclinic, *P*2_1_/*c*	Mo *K*α radiation, λ = 0.71073 Å
Hall symbol: -P 2ybc	Cell parameters from 3289 reflections
*a* = 12.2049 (3) Å	θ = 1.8–28.4°
*b* = 9.8463 (2) Å	µ = 0.11 mm^−^^1^
*c* = 11.5405 (2) Å	*T* = 293 K
β = 107.106 (1)°	Block, colourless
*V* = 1325.51 (5) Å^3^	0.30 × 0.25 × 0.20 mm
*Z* = 4	

### Data collection

**Table:**

Bruker SMART APEXII area-detector diffractometer	3289 independent reflections
Radiation source: fine-focus sealed tube	2644 reflections with *I* > 2σ(*I*)
Graphite monochromator	*R*_int_ = 0.023
ω and φ scans	θ_max_ = 28.4°, θ_min_ = 1.8°
Absorption correction: multi-scan (*SADABS*; Bruker, 2008)	*h* = −13→16
*T*_min_ = 0.969, *T*_max_ = 0.979	*k* = −12→13
12523 measured reflections	*l* = −12→15

### Refinement

**Table:**

Refinement on *F*^2^	Secondary atom site location: difference Fourier map
Least-squares matrix: full	Hydrogen site location: inferred from neighbouring sites
*R*[*F*^2^ > 2σ(*F*^2^)] = 0.041	H atoms treated by a mixture of independent and constrained refinement
*wR*(*F*^2^) = 0.125	*w* = 1/[σ^2^(*F*_o_^2^) + (0.0582*P*)^2^ + 0.2995*P*] where *P* = (*F*_o_^2^ + 2*F*_c_^2^)/3
*S* = 1.06	(Δ/σ)_max_ = 0.004
3289 reflections	Δρ_max_ = 0.26 e Å^−^^3^
195 parameters	Δρ_min_ = −0.18 e Å^−^^3^
0 restraints	Extinction correction: *SHELXL97* (Sheldrick, 2008), Fc^*^=kFc[1+0.001xFc^2^λ^3^/sin(2θ)]^-1/4^
Primary atom site location: structure-invariant direct methods	Extinction coefficient: 0.031 (3)

### Special details

**Table:**

Geometry. All esds (except the esd in the dihedral angle between two l.s. planes) are estimated using the full covariance matrix. The cell esds are taken into account individually in the estimation of esds in distances, angles and torsion angles; correlations between esds in cell parameters are only used when they are defined by crystal symmetry. An approximate (isotropic) treatment of cell esds is used for estimating esds involving l.s. planes.
Refinement. Refinement of F^2^ against ALL reflections. The weighted R-factor wR and goodness of fit S are based on F^2^, conventional R-factors R are based on F, with F set to zero for negative F^2^. The threshold expression of F^2^ > 2sigma(F^2^) is used only for calculating R-factors(gt) etc. and is not relevant to the choice of reflections for refinement. R-factors based on F^2^ are statistically about twice as large as those based on F, and R- factors based on ALL data will be even larger.

### Fractional atomic coordinates and isotropic or equivalent isotropic displacement parameters (Å^2^)

**Table:**

	*x*	*y*	*z*	*U*_iso_*/*U*_eq_	
C1	0.61383 (11)	0.04795 (13)	0.39832 (12)	0.0428 (3)	
C2	0.53048 (13)	0.13768 (17)	0.40961 (15)	0.0577 (4)	
H2	0.4606	0.1437	0.3494	0.069*	
C3	0.55274 (15)	0.21813 (17)	0.51177 (17)	0.0631 (4)	
H3	0.4979	0.2798	0.5202	0.076*	
C4	0.65544 (15)	0.20749 (16)	0.60095 (15)	0.0578 (4)	
H4	0.6694	0.2605	0.6705	0.069*	
C5	0.73827 (12)	0.11780 (14)	0.58743 (12)	0.0469 (3)	
H5	0.8076	0.1114	0.6484	0.056*	
C6	0.72002 (10)	0.03729 (12)	0.48485 (10)	0.0367 (3)	
C7	0.81856 (11)	−0.04361 (12)	0.46565 (11)	0.0383 (3)	
C8	0.87671 (12)	0.30082 (14)	0.40898 (11)	0.0472 (3)	
H8	0.8642	0.2544	0.3360	0.057*	
C9	0.80301 (12)	0.39954 (14)	0.41863 (12)	0.0483 (3)	
H9	0.7407	0.4216	0.3526	0.058*	
C10	0.82151 (11)	0.46900 (13)	0.52995 (12)	0.0429 (3)	
C11	0.91448 (12)	0.43446 (13)	0.62498 (11)	0.0423 (3)	
H11	0.9278	0.4794	0.6987	0.051*	
C12	0.99021 (11)	0.33137 (13)	0.61202 (11)	0.0399 (3)	
C13	0.73825 (14)	0.57626 (16)	0.54149 (15)	0.0597 (4)	
H13A	0.7559	0.6044	0.6246	0.090*	
H13B	0.6618	0.5403	0.5153	0.090*	
H13C	0.7437	0.6528	0.4920	0.090*	
N1	0.58459 (11)	−0.04327 (14)	0.29302 (11)	0.0562 (3)	
N3	0.96840 (10)	0.26833 (11)	0.50367 (9)	0.0417 (3)	
N4	1.08159 (12)	0.29199 (15)	0.70053 (11)	0.0561 (3)	
O1	0.53069 (14)	0.00397 (18)	0.19509 (11)	0.0954 (5)	
O2	0.61421 (12)	−0.16147 (12)	0.30903 (11)	0.0771 (4)	
O3	0.83221 (9)	−0.04027 (11)	0.36362 (9)	0.0547 (3)	
O4	0.88257 (8)	−0.10404 (11)	0.55661 (8)	0.0518 (3)	
H4B	1.0969 (15)	0.3317 (18)	0.7709 (17)	0.059 (5)*	
H4A	1.1221 (15)	0.2174 (19)	0.6872 (16)	0.065 (5)*	
H3A	1.0192 (15)	0.2040 (18)	0.4898 (16)	0.064 (5)*	

### Atomic displacement parameters (Å^2^)

**Table:**

	*U*^11^	*U*^22^	*U*^33^	*U*^12^	*U*^13^	*U*^23^
C1	0.0439 (7)	0.0438 (6)	0.0385 (6)	0.0005 (5)	0.0088 (5)	0.0079 (5)
C2	0.0448 (7)	0.0628 (9)	0.0619 (9)	0.0129 (6)	0.0101 (6)	0.0191 (7)
C3	0.0659 (10)	0.0565 (9)	0.0744 (11)	0.0243 (7)	0.0323 (9)	0.0124 (8)
C4	0.0738 (10)	0.0519 (8)	0.0533 (8)	0.0144 (7)	0.0272 (8)	−0.0016 (6)
C5	0.0513 (7)	0.0510 (7)	0.0379 (6)	0.0061 (6)	0.0122 (6)	−0.0011 (5)
C6	0.0409 (6)	0.0363 (6)	0.0334 (6)	0.0026 (5)	0.0118 (5)	0.0053 (4)
C7	0.0410 (6)	0.0395 (6)	0.0335 (6)	0.0019 (5)	0.0095 (5)	−0.0001 (5)
C8	0.0572 (8)	0.0520 (7)	0.0299 (6)	0.0070 (6)	0.0089 (5)	−0.0020 (5)
C9	0.0504 (7)	0.0528 (7)	0.0368 (6)	0.0087 (6)	0.0053 (5)	0.0017 (5)
C10	0.0460 (7)	0.0396 (6)	0.0435 (7)	0.0020 (5)	0.0139 (6)	0.0008 (5)
C11	0.0503 (7)	0.0417 (6)	0.0352 (6)	0.0010 (5)	0.0130 (5)	−0.0047 (5)
C12	0.0460 (7)	0.0416 (6)	0.0321 (6)	0.0010 (5)	0.0115 (5)	0.0005 (5)
C13	0.0599 (9)	0.0541 (8)	0.0621 (9)	0.0150 (7)	0.0131 (7)	−0.0064 (7)
N1	0.0510 (7)	0.0649 (8)	0.0446 (7)	−0.0082 (6)	0.0016 (5)	−0.0002 (6)
N3	0.0479 (6)	0.0452 (6)	0.0318 (5)	0.0083 (5)	0.0114 (4)	−0.0001 (4)
N4	0.0627 (8)	0.0625 (8)	0.0355 (6)	0.0192 (6)	0.0026 (5)	−0.0050 (5)
O1	0.1008 (11)	0.1144 (12)	0.0473 (7)	0.0141 (9)	−0.0148 (7)	0.0027 (7)
O2	0.1014 (10)	0.0528 (7)	0.0671 (8)	−0.0131 (6)	0.0094 (7)	−0.0113 (6)
O3	0.0669 (7)	0.0634 (6)	0.0391 (5)	0.0162 (5)	0.0237 (5)	0.0062 (4)
O4	0.0522 (6)	0.0648 (6)	0.0372 (5)	0.0209 (5)	0.0111 (4)	0.0079 (4)

### Geometric parameters (Å, º)

**Table:**

C1—C2	1.382 (2)	C9—C10	1.4137 (19)
C1—C6	1.3880 (17)	C9—H9	0.9300
C1—N1	1.4682 (18)	C10—C11	1.3688 (18)
C2—C3	1.379 (3)	C10—C13	1.4985 (19)
C2—H2	0.9300	C11—C12	1.4101 (18)
C3—C4	1.372 (2)	C11—H11	0.9300
C3—H3	0.9300	C12—N4	1.3295 (17)
C4—C5	1.385 (2)	C12—N3	1.3505 (16)
C4—H4	0.9300	C13—H13A	0.9600
C5—C6	1.3872 (18)	C13—H13B	0.9600
C5—H5	0.9300	C13—H13C	0.9600
C6—C7	1.5122 (17)	N1—O2	1.2165 (18)
C7—O3	1.2372 (15)	N1—O1	1.2203 (17)
C7—O4	1.2585 (15)	N3—H3A	0.933 (18)
C8—C9	1.3510 (19)	N4—H4B	0.871 (19)
C8—N3	1.3526 (17)	N4—H4A	0.923 (19)
C8—H8	0.9300		
			
C2—C1—C6	122.54 (13)	C10—C9—H9	120.3
C2—C1—N1	117.55 (13)	C11—C10—C9	118.75 (12)
C6—C1—N1	119.83 (12)	C11—C10—C13	121.87 (12)
C3—C2—C1	118.83 (14)	C9—C10—C13	119.37 (12)
C3—C2—H2	120.6	C10—C11—C12	120.57 (12)
C1—C2—H2	120.6	C10—C11—H11	119.7
C4—C3—C2	120.27 (14)	C12—C11—H11	119.7
C4—C3—H3	119.9	N4—C12—N3	118.01 (12)
C2—C3—H3	119.9	N4—C12—C11	123.81 (12)
C3—C4—C5	120.01 (15)	N3—C12—C11	118.18 (11)
C3—C4—H4	120.0	C10—C13—H13A	109.5
C5—C4—H4	120.0	C10—C13—H13B	109.5
C4—C5—C6	121.40 (13)	H13A—C13—H13B	109.5
C4—C5—H5	119.3	C10—C13—H13C	109.5
C6—C5—H5	119.3	H13A—C13—H13C	109.5
C5—C6—C1	116.90 (11)	H13B—C13—H13C	109.5
C5—C6—C7	119.41 (11)	O2—N1—O1	124.08 (15)
C1—C6—C7	123.29 (11)	O2—N1—C1	118.07 (12)
O3—C7—O4	125.59 (12)	O1—N1—C1	117.84 (14)
O3—C7—C6	117.46 (11)	C12—N3—C8	122.02 (11)
O4—C7—C6	116.88 (10)	C12—N3—H3A	120.8 (11)
C9—C8—N3	121.08 (12)	C8—N3—H3A	117.1 (11)
C9—C8—H8	119.5	C12—N4—H4B	119.2 (11)
N3—C8—H8	119.5	C12—N4—H4A	118.3 (11)
C8—C9—C10	119.39 (12)	H4B—N4—H4A	122.2 (16)
C8—C9—H9	120.3		
			
C6—C1—C2—C3	−1.2 (2)	N3—C8—C9—C10	−0.6 (2)
N1—C1—C2—C3	175.64 (14)	C8—C9—C10—C11	0.4 (2)
C1—C2—C3—C4	−0.8 (2)	C8—C9—C10—C13	−178.59 (14)
C2—C3—C4—C5	1.4 (3)	C9—C10—C11—C12	−0.1 (2)
C3—C4—C5—C6	0.0 (2)	C13—C10—C11—C12	178.84 (13)
C4—C5—C6—C1	−1.9 (2)	C10—C11—C12—N4	−179.53 (14)
C4—C5—C6—C7	171.12 (13)	C10—C11—C12—N3	0.1 (2)
C2—C1—C6—C5	2.48 (19)	C2—C1—N1—O2	−137.16 (16)
N1—C1—C6—C5	−174.27 (12)	C6—C1—N1—O2	39.75 (19)
C2—C1—C6—C7	−170.21 (12)	C2—C1—N1—O1	41.9 (2)
N1—C1—C6—C7	13.05 (18)	C6—C1—N1—O1	−141.19 (15)
C5—C6—C7—O3	−134.09 (13)	N4—C12—N3—C8	179.29 (13)
C1—C6—C7—O3	38.42 (18)	C11—C12—N3—C8	−0.33 (19)
C5—C6—C7—O4	43.01 (17)	C9—C8—N3—C12	0.6 (2)
C1—C6—C7—O4	−144.48 (13)		

### Hydrogen-bond geometry (Å, º)

**Table:**

*D*—H···*A*	*D*—H	H···*A*	*D*···*A*	*D*—H···*A*
N3—H3*A*···O4^i^	0.933 (18)	1.752 (19)	2.6746 (16)	169.5 (17)
N4—H4*A*···O3^i^	0.923 (19)	1.972 (19)	2.8734 (18)	164.9 (17)
N4—H4*B*···O4^ii^	0.871 (19)	2.033 (19)	2.8937 (16)	169.7 (18)
C11—H11···O3^iii^	0.93	2.58	3.3624 (16)	142

Symmetry codes: (i) −*x*+2, −*y*, −*z*+1; (ii) −*x*+2, *y*+1/2, −*z*+3/2; (iii) *x*, −*y*+1/2, *z*+1/2.
